# Review of Prognosis Approaches Applied to Power SiC MOSFETs for Health State and Remaining Useful Life Prediction

**DOI:** 10.3390/e28020234

**Published:** 2026-02-17

**Authors:** Sanjiv Kumar, Bruno Allard, Malorie Hologne-Carpentier, Guy Clerc, François Auger

**Affiliations:** 1 INSA Lyon, Universite Claude Bernard Lyon 1, Ecole Centrale de Lyon, CNRS Ampère, UMR5005, 69621 Villeurbanne, France; bruno.allard@insa-lyon.fr (B.A.); guy.clerc@univ-lyon1.fr (G.C.); 2 LabECAM, ECAM LaSalle, 69005 Lyon, France; malorie.carpentier@ecam.fr; 3 IREENA, Nantes Université, UR 4642, 44600 Saint-Nazaire, France; francois.auger@univ-nantes.fr

**Keywords:** silicon carbide (SiC) MOSFETs, power electronic, prognosis, predictive maintenance, failure prediction, Remaining Useful Life, condition monitoring, reliability, data-driven methods, neural networks

## Abstract

The use of Silicon Carbide (SiC) MOSFETs significantly improves converter performance by increasing efficiency and reducing costs, to the detriment of electro-magnetic emission and reliability. Implementing a predictive maintenance strategy based on a prognosis tool can mitigate this limitation. This literature review offers a methodological synthesis of prognosis design tools for SiC MOSFETs, while also encompassing studies on IGBTs and silicon-based power MOSFETs where these approaches are transferable. The analysis focuses on wear-out prognosis under nominal operating conditions of standard package device, excluding environmental constraints. Articles published up to 2025 were identified in the OpenAlex database using a keyword-based search and manually filtered according to the study scope. Most reviewed works rely on Data-Based prognosis methods, mostly based on neural networks, though out-of-sample validation remains uncommon. Our study also highlights the dependence of Data-Based prognosis performance on the shape of degradation indicator trends. Moreover, the estimation of prediction uncertainty is rarely addressed in the reviewed literature. Despite notable methodological advances, ensuring the reliability of prognosis tools for SiC MOSFETs remains an ongoing research challenge.

## 1. Introduction

The electrification of transportation and industrial sectors, coupled with the expansion of renewable energy systems, represents a cornerstone of the global energy transition. The ecology and energy transitions are driven by the urgent need to mitigate climate change and to reduce the dependence of the economies on fossil fuels [1]. Achieving large-scale electrification requires the deployment of advanced power electronic converters capable of handling and regulating medium to high direct voltages, systems inherently more complex than conventional transformers. However, the widespread deployment of power converters introduces significant challenges in terms of system cost, efficiency and reliability. Studies reported in [2,3], based on the analysis of field data collected from 350 photovoltaic farms over a 27-month period, indicate that approximately 6.5 GWh of energy was not produced due to power plant failures. Among the identified failure sources, inverters were found to account for 43% of the observed operational failures in photovoltaic power plants. Moreover, inverter failures were responsible for 36% of the total energy production losses, making them the primary contributor to reduced productivity in photovoltaic systems. Converter failures and losses are predominantly attributed to the active semiconductor devices integrated within the converters. Investigations gathered in [4] have shown that more than 30% of system faults originate from the active components.

In 2011, Cree Inc. introduced the first commercial silicon carbide (SiC) power MOSFET, marking a pivotal milestone in the development of wide bandgap (WBG) power semiconductor technologies. SiC MOSFETs exhibit several advantages over conventional silicon (Si) devices, including high breakdown voltage, operation at higher junction temperature, greater power handling capability and fast switching speed [5]. These characteristics enable SiC devices to address key challenges in efficiency and cost reduction for modern power electronic converters. Despite these advantages, SiC MOSFETs still exhibit lower reliability levels compared with traditional Si IGBT devices [6,7,8]. The limitation is primarily attributed to the increased complexity of SiC manufacturing processes and the intrinsic physical properties of WBG materials. Consequently, SiC devices tend to amplify the existing reliability concerns associated with power electronic converters.

From a user’s perspective, adopting a planned maintenance strategy is an effective approach to ensure the operational safety of a system that exhibits robustness issues [9]. In industrial practice, preventive maintenance is often the preferred method. It consists of scheduling the replacement of critical components at regular intervals, according to the manufacturer’s recommendations. However, given the economic cost of power semiconductor devices and the environmental impact associated with their production [10,11], such maintenance strategy is not fully aligned with economic efficiency and sustainable development goals, because the considered critical component is replaced regardless of its actual degradation state. Consequently, there is a growing interest in predictive maintenance methods. This approach relies on the use of a prognosis tool, capable of estimating the future degradation state of a component based on its current condition, thereby enabling the assessment of its Remaining Useful Life (RUL). Estimating the RUL of a component enables the scheduling of maintenance actions while maximizing its operational lifetime and minimizing unnecessary replacements. Beyond maintenance scheduling, accurate RUL prognosis can also be leveraged at the system level to further enhance its lifetime. By integrating health information into control and supervision strategies, the operating conditions of the device can be adapted according to its degradation state. Such health-aware control approaches contribute to extending not only the remaining lifetime of individual devices, but also the operational lifetime and availability of the overall system, while maintaining acceptable performance levels.

Developing a prognosis tool is a complex and inherently multidisciplinary task [9]. Nevertheless, such a tool is a relevant way to address reliability challenges in power electronic converters, thereby supporting the ongoing energy transition. As a result, the scientific community has shown growing interest in this research field over the past decade. Although several studies have already provided reviews of prognosis methods applied to SiC MOSFETs and/or IGBTs [12,13,14,15], they generally address the topic from a technical or algorithmic perspective, without identifying broader methodological trends. Furthermore, the critical analyses provided by authors proposing prognosis methods tend to concentrate on the technical features of their tools, while offering limited consideration on the methodological implications of their potential integration into industrial maintenance frameworks. Consequently, this paper presents a review of prognosis methods for SiC MOSFETs, now limited in application to medium voltage dices, highlighting key methodological trends and contextualizing their relevance to the improvement of industrial maintenance systems. Most existing studies on prognosis techniques have focused primarily on Si IGBTs and Si power MOSFETs. On the one hand, research on IGBT prognosis has a longer history, and in many comparable applications, IGBTs remain more widely adopted than SiC MOSFETs. On the other hand, the release of an accelerated ageing dataset of IGBT and Si power MOSFET from the NASA Prognostics Center of Excellence (PCoE) [16,17] has significantly contributed to the increase in the number of related publications. Although SiC MOSFET chips exhibit specific failure mechanisms, both IGBTs, Si power MOSFETs and SiC MOSFETs share similar packaging-related degradation modes [6,7,8,18]. Consequently, many prognosis methods originally developed for IGBTs or Si power MOSFETs can be adapted and applied to SiC MOSFETs. Therefore, this review of SiC MOSFET prognosis methods also encompasses approaches originally developed for IGBTs or Si power MOSFETs, insofar as these methods are potentially applicable to SiC technologies. The analysis focuses on wear-out prognosis under nominal operating conditions of devices in standard package, excluding environmental constraints. Articles published up to 2025 were retrieved from the OpenAlex database through a keyword-based search and subsequently filtered manually to align with the scope of this study. The paper presents a synthesis of the reviewed literature database, further enriched by the authors’ expertise in power semiconductor components and RUL prediction.

The structure of this paper is as follows. Section 2 outlines the foundation principles and general methodological framework guiding the design of a prognosis tool. Section 3 presents a comprehensive review of the lifetime estimation techniques reported in the literature. Section 4 provides a critical assessment of the practical relevance and constraints associated with these methodologies. Finally, a conclusion to this work is presented outlining key insights of the proposed review.

## 2. Development of a Prognosis Tool

### 2.1. Standard Methodology

**Figure 1:**
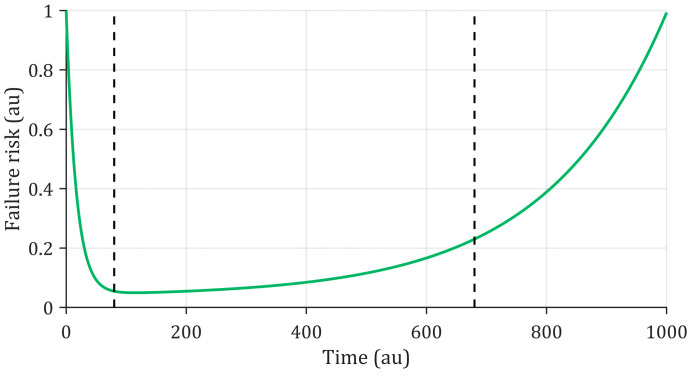
Bathtub curve (au: arbitrary unit).

The evolution of the failure risk of a component operating within its Safe Operating Area (SOA) is traditionally represented by a bathtub curve [9] as illustrated in Figure 1.

At the beginning of the component’s life, the failure risk is high, mainly due to manufacturing or design defects. Such occurrences are referred to as early failures or infant mortality failures, and are generally assigned to the manufacturer’s responsibility, often stemming from latent design or production flaws. Following this initial period, the failure risk decreases and remains relatively stable during the component’s normal operational phase. Failures occurring at this stage are not caused by the component itself but typically result from external environmental factors, leading to extrinsic failures. Such failures are therefore unpredictable at the component level and are often modeled as random processes. Finally, the failure risk increases over time due to component aging. Component aging is typically assumed to progress gradually and is predominantly influenced by the prevailing operating conditions that generate stressors. Consequently, at the scale of the component and the end user, the development of a prognosis tool may primarily focus on exclusively studying the component’s aging process to estimate its RUL.

However, the expected lifetime of a SiC MOSFET operating within its SOA is typically on the order of ten years or more. Analyzing the wear-out behavior of such devices over extended timescales is both challenging to execute and of limited practical significance. Indeed, the technological development cycle of SiC MOSFETs progresses faster than their theoretical operational lifetime. Since their introduction in the early 2010s, SiC MOSFETs have undergone significant advancements, reaching their fourth generation by 2025 [19]. Consequently, by the end of a hypothetical long-term aging study, the collected results would inevitably correspond to a now obsolete generation of SiC MOSFETs.

**Figure 2:**
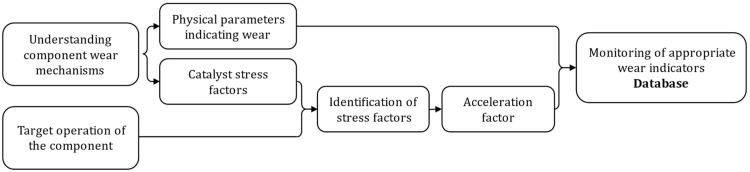
Typical accelerated aging test methodology.

In this context, the traditional approach used to study component wear-out involves conducting an accelerated aging test campaign. The general methodology of such a campaign is illustrated in Figure 2.

At the initial level, the goal is to grasp the physics of failure governing the component to identify its underlying degradation mechanisms. The purpose of the analysis is twofold: first, to determine physical parameters that are indicative of the component’s degradation state, and second, to identify the stress factors that contribute to the acceleration of the wear-out process. At a second level, the target operating conditions of the component must be carefully defined to identify the main stress factors it will encounter in real world applications. If the system is properly designed, the stress factors generally have low amplitude and/or low occurrence frequency, leading to component degradation only over several years of operation. To successfully conduct an accelerated aging campaign, it is therefore necessary to increase the amplitude and/or raise the occurrence frequency of the identified stress factors. The latter process is commonly known as the application of an acceleration factor. During the test phase, a set of parameters indicative of the component’s degradation state is continuously measured. The collected measurements, along with their associated test conditions, constitute an accelerated aging database that serves as a foundation for validating or developing a prognosis method.

The key aspect of an accelerated aging campaign lies in the selection of the acceleration factor. This choice represents a critical assumption about the extent to which accelerated aging data accurately reflects the actual, yet unknown, long-term degradation behavior. Indeed, the chosen acceleration factor must not introduce failure mechanisms that would not occur under normal operating conditions. Traditionally, the acceleration factor is derived from physical considerations expressed as a function of a specific stress feature. Classic examples include the Arrhenius law, where the temperature serves as the acceleration factor, and the Coffin-Mason law, which models thermal fatigue using temperature variation as the acceleration parameter [20].

### 2.2. Failure Modes of SiC MOSFETs

As discussed in the previous section, implementing a prognosis method requires a thorough understanding of the component’s failure physics to design a relevant accelerated aging campaign. Comprehensive review of SiC MOSFET degradation mechanisms can already be found in the literature [6,7,8]. This section therefore provides a summary of the main failure mechanisms affecting these devices.

**Figure 3:**
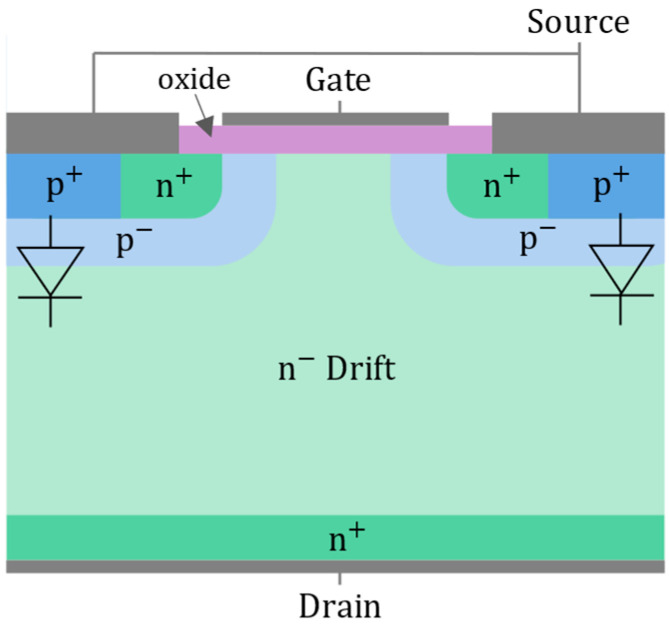
Typical cross-sectional view of an n-type vertical diffused SiC MOSFET.

Power SiC MOSFETs adopt a vertical architecture [21]. Figure 3 shows a typical cross-sectional view of an n-type vertical diffused SiC power MOSFET. This structure consists of a heavily doped substrate, a lightly doped drift region, and a gate electrode insulated by an oxide layer that controls the current flow between drain and source. Such a configuration offers low on-state resistance, high breakdown voltage capability and an integrated body diode.

At the chip level, the most critical regions are the gate oxide [22] and the body diode [23]. The gate oxide degrades due to tunneling currents passing through it, which is a phenomenon particularly significant in SiC MOSFETs [24,25]. On the one hand, the oxide growth process introduces defects and impurities within the crystal lattice, creating intermediate energy states between the conduction and the valence bands, which facilitates carrier tunneling [26,27,28]. On the other hand, the WBG nature of SiC materials reduces the band offsets between the semiconductor and the oxide, further promoting carrier transport through Fowler–Nordheim tunneling [24,25,29]. Additionally, the electron mobility in SiC is lower than in Si, requiring a thinner oxide layer to maintain reasonable gate voltages, which inherently makes the oxide less robust [30,31]. The body diode is primarily degraded by the energy released during carrier recombination at the PN junction. This energy can generate or expand stacking faults in the semiconductor crystal lattice, leading to performance degradation over time [23,32,33].

**Figure 4:**
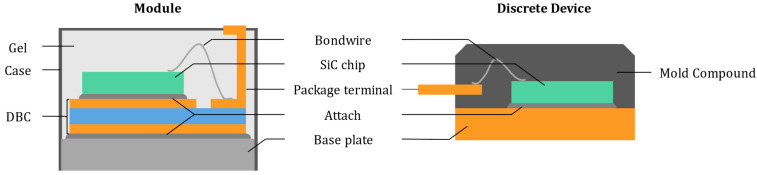
Internal structure of a module and a discrete device.

The semiconductor die is assembled within a package, to form either a discrete device or a module. A discrete device package contains a single die, while a module integrates multiple chips, enabling operation at higher current and voltage levels. Modules also include an insulated baseplate, simplifying integration within power converters. Figure 4 illustrates the internal structure of these packages.

In modules, the die is soldered or sintered onto a Direct Bonded Copper (DBC) substrate, which is itself attached to the baseplate. The DBC consists of alternating layers of copper-dielectric-copper, providing both electrical insulation and efficient heat dissipation. In discrete devices, the die is directly soldered or sintered onto a copper (Cu) baseplate [34]. The joints between the die and the baseplate are referred to as solder layers. Bond wires, typically made of aluminum (Al), connect the metallized terminals of the die to the external package leads through ultrasonic bonding [35]. This assembly process is generally referred to as the bonding-solder technology. In discrete devices, the entire assembly is encapsulated in a mold compound, ensuring mechanical rigidity. In power modules, the internal structure is embedded in a dielectric gel, while mechanical integrity is ensured by the external casing.

Within the standard packages, the most failure-prone areas are the metallic interfaces, namely the chip-bond wire connection and the various solder layers. Cu, Al and SiC have different Coefficients of Thermal Expansion (CTE), which lead to significant mechanical stresses at these interfaces during temperature cycling. At the chip-bond wire interface, the accumulation of these thermomechanical stresses induces crack formation, potentially resulting in complete bond wire lift-off [35,36,37]. Similarly, at the solder interfaces, repeated stress leads to delamination, reducing the thermal dissipation capability of the chip [20,38].

The assembly process is similar for IGBTs and Si power MOSFETs. Therefore, IGBTs, Si power MOSFETs and SiC MOSFETs share the same packaging-related failure modes, though their degradation dynamics may differ. Though the CTE mismatch between SiC and Al/Cu is slightly lower than that between Si and the same metals, SiC material higher Young’s modulus makes it stiffer and thus more prone to stronger thermomechanical stress [39]. Nonetheless, prognosis methods related to temperature cycling-induced degradation are generally transferable between the two technologies.

More robust packaging technologies, such as press-pack or double-sided-cooled architectures, do exist. However, most commercially available devices still rely on the bonding-solder structure described above. As a result, this review concentrates solely on the latter packaging technology, although studies on degradation and prognosis for alternative assemblies are also available in the literature [40,41,42,43]. Multiple discrete packaging structures, such as TO-247-4 and D2PAK, exist. As long as they rely on a bonding-solder architecture, they are subject to similar overall wear-out mechanisms, although the wear-out dynamics may differ. For instance, surface-mount device packages such as D2PAK generally present greater thermal management challenges, which can affect the device degradation rate.

Table 1, adapted from the synthesis presented in [7], outlines the main SiC MOSFET failure mechanisms, their causes, the stress factors accelerating their occurrence, and the key physical indicators representative of the degradation state of each region.

Additional failure mechanisms related to environmental conditions, such as humidity or exposure to radiation, also exist [44,45,46,47]. However, these aspects are not addressed in the present review.

### 2.3. Accelerated Aging and Typical Drift of Degradation Indicators

**Figure 5:**
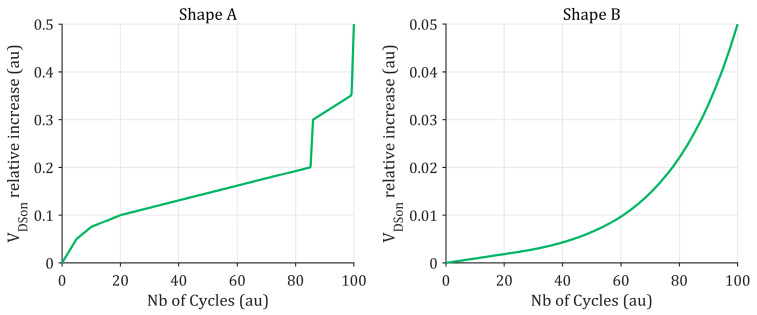
Sketch of different degradation behavior patterns reported in the literature during thermal cycling.

Temperature cycling of a component is an unavoidable stress, even when the system design complies with the component’s SOA limits. As a result, most published studies rely on accelerated thermal cycling tests to construct or validate prognostic models. Several approaches exist to perform such cycling. Most studies rely on active power cycling, where temperature variations within the component are driven by its own power dissipation [48,49,50,51]. In this context, the most measured degradation indicators are the on-state voltage or the on-state resistance. Regardless of the cycling conditions, the evolution of these indicators can take various shapes, as illustrated in Figure 5.

Monotonic drifts are generally attributed to crack formation at the chip-bond wire interface and to gate oxide degradation in SiC MOSFETs, which leads to threshold voltage shifts and corresponding changes in channel resistance. In contrast, abrupt and highly nonlinear variations are typically linked to bond wire lift-off. Although Shape B appears more predictable, it should not be interpreted as an improvement over Shape A. Both shapes illustrate possible outcomes of on-state voltage evolution reported in prior studies. While further investigation would be required to explain the origins of these differing behaviors, the goal is to emphasize that the performance of prognosis tools may vary depending on the underlying degradation indicator trend, as discussed in the remainder of the paper.

Other and less common studies in the field of prognostics focus on developing methods specifically designed to assess the health status of the gate oxide in SiC MOSFETs. Traditional accelerated aging tests in this case involve applying high voltage and temperature stress to the oxide. The High Temperature Reverse Bias (HTRB) test is widely used in the literature. It involves applying a strongly negative gate-source potential while maintaining the device in a high-temperature chamber [52,53,54]. Under the latter conditions, the threshold voltage and the gate leakage current are typically measured.

A few works also address degradation of the body diode [55], although these are much rarer. In practice, the degradation of the body diode is rarely considered in prognosis methods, since SiC MOSFETs used in power converters are often paired with an external antiparallel diode that carries most of the reverse current.

Regardless of the selected aging protocol, the test may be carried out either until component failure or until a monitored degradation indicator exceeds a predefined threshold. For instance, as specified in the AQG 324 standards [56], a device is considered to have failed when its on-state voltage exhibits a 5% increase or when the chip-to-baseplate thermal resistance rises by 20%. The cycle at which the component reaches this failure condition is referred to as $N_{f}$. The RUL is modeled as a monotonically decreasing linear function, starting at 1 for a new component and reaching 0 upon failure. Given $N_{f}$, the RUL as a function of the current cycle N can be expressed according to Equation (1).

(1) $$RUL\left(N\right)=1-\frac{N}{N_{f}}$$

## 3. Prognosis Methods

Prognosis methods are traditionally classified into three categories: Physics-Based, Data-Based, and Hybrid. Physics-Based methods rely on the formulation of a lifetime model that describes the progression of a specific failure mechanism. Data-Based prognosis tools rely exclusively on accelerated aging data and algorithmic approaches, without incorporating explicit physical models. Hybrid strategies bridge the gap between physics-based and data-driven methodologies, aiming to leverage the complementary advantages of each one.

### 3.1. Physics-Based Methods

#### 3.1.1. General Considerations

**Figure 6:**
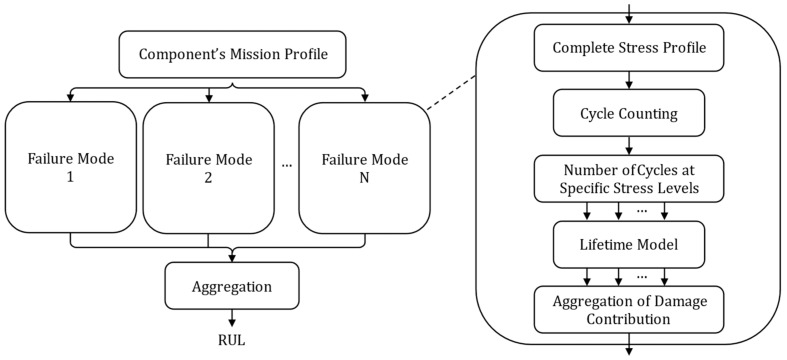
Simplified Schematic of the Physics of Failure framework.

These models most often express the number of cycles to failure of a component as a function of physical parameters, either estimated or measured at the component level. The use of such lifetime models is typically framed within the Physics of Failure (PoF) concept [57,58,59,60,61,62,63,64,65,66,67], as illustrated in Figure 6.

Within this framework, component prognosis relies exclusively on the characterization of environmental stresses, the stress profile, to which the component is exposed. The full complex stress profile is decomposed using cycle-counting methods such as the *Rainflow* algorithm [68] into a number of cycles at specific stress levels. For each failure mode of the component, a lifetime model is updated based on the number of cycles at the corresponding stress levels. These contributions are then combined, mostly linearly according to Miner’s cumulative damage law. The outputs of the different degradation models are then aggregated to estimate the RUL of the component. The effectiveness of these methods depends on the accuracy of the stress profile assessment, the modeling of damage accumulation and failure mode interactions, and the choice of lifetime model. Lifetime models are generally based on empirical considerations and can be divided into two categories: physical models [69,70,71,72] and analytical models [73,74,75,76]. The following sections provide a detailed overview of the two approaches.

#### 3.1.2. Physical Lifetime Model

Physical lifetime models characterize degradation mechanisms based on physical principles associated with the targeted failure mode. The number of cycles to failure is typically expressed as a function of direct physical stresses, which are generally not measurable at the component level. Consequently, the design of such models relies mostly on Finite Element Method (FEM) simulations. Accurate simulation relies on an in-depth knowledge of the component’s physical architecture, which is frequently challenging to access or model precisely. Moreover, as discussed in [20], FEM numerical simulations are subject to geometric singularities, which can introduce errors in the estimation of local physical stresses. Accelerated aging tests are employed to validate or refine the constructed lifetime model.

Most physical lifetime models reported in the literature primarily address the degradation of solder layers and the chip-to-bonding wire interface. A comprehensive review of these models is provided in [77]. The purpose of this section is therefore to present a synthetic overview of the latter approaches, emphasizing several trends observed in the literature. According to the classification proposed in [78], these models can be divided into four main categories, although, in practice, most models combine features from multiple categories [77]: strain-based model, energy-based model, creep damage-based model and damage accumulation-based model.

Strain-based models are the most used. Plastic strain is identified as the dominant damage mechanism in these modeling approaches. They establish a relationship between the lifetime in number of cycles to failure and the imposed cyclic strain amplitude. The Coffin–Manson model [70] in Equation (2) is traditionally employed for this purpose:

(2) $$N_{f}={\left(\frac{C}{\Delta \varepsilon_{p}}\right)}^{\frac{1}{m}}$$

where $N_{f}$ is the number of cycles to failure, $\Delta \varepsilon_{p}$ represents the plastic strain range, m is the fatigue exponent, and C is the ductility coefficient. For instance, this model is employed in [79], that proposes a digital twin of a SiC power module to predict its lifetime. In [80], the model is refined to correlate $N_{f}$ with a given characteristic crack length. Subsequently, reference [63] further improves the model to insure that the characteristic crack length aligns with the failure threshold values of degradation indicators defined by the AQG 324 standards [56]. Engelmaier et al. in [72] proposed in Equation (3) a more sophisticated version of the Coffin–Manson model by incorporating both the cycling frequency and the temperature of the solder layer:

(3) $$N_{f}=\frac{1}{2}{\left(\frac{\Delta \gamma }{2\epsilon_{f}^{\prime }}\right)}^{\frac{1}{c}}$$

where Δγ represents the cyclic shear strain range, and $\epsilon_{f}^{\prime }$ denotes the fatigue ductility coefficient. The fatigue ductility exponent c is determined as a function of the cycling frequency and the average temperature of the solder layer, thereby capturing the combined thermo-mechanical effects on material fatigue behavior.

Similarly, energy-based models are also frequently employed. In the latter approaches, the amount of dissipated energy, most often plastic or viscoplastic, is considered as the primary damage variable. The underlying assumption is that energy dissipation reflects the material’s cumulative damage capacity. The Morrow model in [71] in Equation (4) is traditionally used:

(4) $$N_{f}={\left(\frac{C}{W_{p}}\right)}^{\frac{1}{m}}$$

where m is the fatigue exponent, C the material ductility coefficient, and $W_{p}$ the plastic strain energy density for the steady-state loop. The strain energy varies with the degradation state of the solder layer. Several studies [81,82,83] propose refined models that incorporate solder layer degradation to adjust cyclic energy accordingly. For instance, reference [82] assesses the deformation energy of the component through FEM simulations under varying thermal resistance values of an IGBT module. The energy value is then updated as the component ages. To reduce the computational cost associated with FEM simulations, several works employ the so-called Clech algorithm, which estimates equivalent stress and strain amplitudes in the solder joints based on a simplified analytical model of their thermomechanical behavior [84,85,86]. These values can then be used to compute the plastic energy dissipated per cycle and predict the component’s lifetime using Morrow-type models. For example, reference [84] refines the algorithm’s coefficients through correlation with FEM results to improve calculation accuracy while minimizing computational resources.

Creep damage-based models are less commonly employed. In these models, a creeping phenomenon is identified as the primary damage mechanism, describing the slow deformation of a metallized interface under sustained stress and elevated temperature [87,88].

Finally, damage accumulation-based models could be mentioned. The models assume that the total damage results from the integration of partial damages occurring during each cycle or loading phase. Reference [77] further classifies the latter models into linear damage accumulation approaches, nonlinear damage accumulation approaches, and crack initiation and propagation approaches. Among linear damage accumulation approaches, the previously mentioned Miner’s rule is an example. In crack propagation approaches, many authors adopt the Paris law [69] as expressed in Equation (5), sometimes modified, to model crack growth along the chip-bond wire interface [36,89,90,91]:

(5) $$\frac{dl}{dN}=c_{1}{\left(\Delta K\right)}^{c_{2}}$$

where N is the number of cycles, l is the crack length, ΔK is the strain intensity factor at the crack tip and $c_{1}$, $c_{2}$ are material constants. In particular, references [36,91] propose an analytical model of the electrical resistance of the chip-bond wire interface as a function of the crack length. This allows the non-destructive estimation of the crack length from the measured on-state voltage of the component, which can then be used with the Paris law to evaluate $N_{f}$.

#### 3.1.3. Analytical Lifetime Model

Extracting strain or energy components from physical lifetime models depends on device operating conditions and FEM simulations, which are computationally intensive, time-consuming, and require detailed knowledge of the component’s architecture. Therefore, analytical lifetime models aim to represent degradation behavior through indirect physical stress parameters that are more accessible to experimental measurements. Like physical models, most analytical lifetime models focus on thermo-mechanical stresses in device packaging, especially the degradation of bond wires and solder layers. All reported models rely on temperature measurements to represent physical stress. Over time, models of varying levels of accuracy and complexity have been proposed. The most commonly used models traditionally referenced in the literature are listed in [92,93]. Like physical lifetime models, the analytical models rely on a set of constant parameters, estimated from simulation results or experimental data. The full formulation of the Bayerer model is detailed in Table 2.

To conduct accelerated lifetime tests within a reasonable timescale, thermal cycling tests are typically performed using large temperature swings, as the temperature swing is considered as a key acceleration factor. Consequently, most proposed lifetime models have been developed based on thermal cycling experiments conducted under large temperature swing conditions, though such swing rarely occur in real applications. For instance, the Bayerer model [75] was derived for $\Delta T_{j}$ values greater than 40 °C. Several studies have extrapolated the applicability of such a model to lower $\Delta T_{j}$ ranges [61,65]. However, references [94,95] demonstrated a deviation of this model under low $\Delta T_{j}$ conditions through dedicated accelerated aging tests. Under small $\Delta T_{j}$, the induced strains are rather elastic than plastic, which explains the observed deviation, according to the authors. To account for this transition to the elastic regime, reference [94] proposes to model the temperature swing exponent in the Bayerer model ($a_{2}$ in the Bayerer expression in Table 2) as a function of $\Delta T_{j}$.

#### 3.1.4. Other Physics Approaches

Although PoF methods represent many Physic-Based approaches, alternative modeling strategies also exist. These are primarily empirical models derived from observations and/or physical considerations to describe the observed variations in degradation indicators. For example, references [96,97,98] propose power-law models, with varying levels of sophistication, to capture the evolution of the threshold voltage of SiC MOSFETs subjected to gate-oxide stress tests. However, these degradation models are rarely used alone. They most often serve as the foundation for parametric Data-Based methods, that are discussed in the following section.

### 3.2. Data-Based Methods

#### 3.2.1. General Considerations

Data-Based prognosis methods are approaches developed directly from the analysis of component aging data. The implementation of such methods does not require *a priori* knowledge of the component’s physical behavior. By modeling aging exclusively through the evolution of degradation indicators, the latter approaches generally outperform physics-based methods, especially when multiple degradation mechanisms are present. However, their effectiveness strongly relies on the quantity and quality of available data. Moreover, as these methods are not based on physical principles, they often lack interpretability, hindering the establishment of a direct connection between observed degradation patterns and the underlying physical failure mechanisms.

#### 3.2.2. Algorithmic Approaches

**Figure 7:**
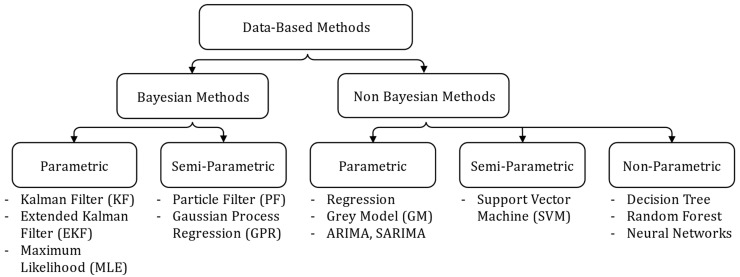
Main algorithms used for constructing Data-Based prognosis approaches.

The main algorithmic tools used in the literature to implement Data-Based prognosis methods are presented in Figure 7.

The presented algorithms are based on machine learning concepts. These algorithms can be divided into two main categories: derived from Bayesian theory or not. The Bayesian theory relies on the concept of knowledge updating: a prior probability, representing the initial understanding of a quantity or component of interest, is updated using new related information to produce a posterior probability:

(6) $$P\left(\theta |D\right)=\;\frac{P\left(D|\theta \right)P(\theta )}{P(D)}$$

where $P\left(\theta |D\right)$ (Posterior) is the updated probability of a parameter θ after observing data D, $P\left(D|\theta \right)$ (Likelihood) is the probability of observing D given θ, P(θ) (Prior) is the initial belief about θ before considering data D, and P(D) (Marginal Likelihood) is the overall probability of the observed data D. Thus, the posterior probability provides an update estimate that reflects the most recent information available about the system. Non-Bayesian methods, in contrast, are founded on alternative principles, most notably the optimization of a cost function. The classification can be further refined by distinguishing among parametric, semi-parametric, and non-parametric approaches. Parametric methods are based on a predefined function form characterized by a finite set of parameters to be estimated. In contrast, non-parametric methods do not assume any fixed function structure: the model adapts directly to the underlying data distribution. Semi-parametric methods combine both paradigms, integrating a parametric core structure with a non-parametric component, offering enhanced modeling flexibility while retaining elements of known structural behavior.

The functional form of parametric methods typically reflects an assumed evolution of a degradation indicator. This function is commonly known as a degradation model and is often constructed from empirical observations. The degradation model can be extended to incorporate random phenomena inherent in the indicator’s variability, resulting in what is known as a stochastic parametric model. A widely used stochastic model for representing degradation processes is the Wiener process [99,100,101]. This model adds Brownian noise to the empirical degradation trend to capture random fluctuations. The performances of parametric methods are largely conditioned by the choice of the degradation function. Since non-parametric methods rely exclusively on training data, they often suffer from limited interpretability and their accuracy strongly depends on the quantity and quality of the available training dataset.

Non-Bayesian methods are widely used, especially non-parametric and semi-parametric approaches. Their popularity stems from their reliance on modern artificial intelligence algorithms, a major focus of current research. The IGBT and Si power MOSFET accelerated aging database published online by PCoE [16,17] has helped draw interest from reliability and data analysis researchers, beyond just electrical engineers, leading to the use of advanced learning-based methods for power transistor prognosis. These algorithms can capture complex and nonlinear relationships, enabling the establishment of a relation between the drift of degradation indicators and component aging. These methods exhibit excellent performances when the training dataset is large and of high quality with respect to the application domain of the algorithm. The latter category of methods includes Support Vector Machine (SVM) and related variants [53,101,102,103,104,105], Decision Tree [104], or Random Forest [53,104,106]. SVM is a supervised learning algorithm that seeks an optimal hyperplane to separate data points through successive projections into higher-dimensional spaces. The Decision Tree is a supervised learning method that splits data into subsets based on simple decision rules, forming a tree-like structure where each leaf represents a final decision or prediction derived from the input features. The Random Forest combines multiple Decision Trees to enhance robustness and generalization. The latter algorithms are applicable for both regression and classification purposes. However, neural networks remain the predominant choice in most published works on prognosis tools so far.

**Figure 8:**
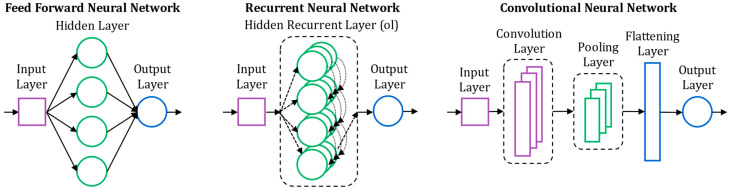
Schematic representation of main neural network architectures reported in the literature (ol: output last).

Thanks to their highly nonlinear architecture and advanced learning mechanisms, neural networks generally exhibit excellent performance with good generalization capabilities for modelling complex systems. However, the inherent high performance comes with significant computational demands, due to numerous trainable parameters and the need for larger training datasets compared to traditional machine learning methods. Hyperparameter optimization can also be tedious and resource-intensive. Various neural network architectures and cell types have been reported in the literature. Some schematic representations are provided in Figure 8.

Several authors use Feedforward Neural Networks (FNN) [107,108,109,110,111,112,113], which are conventional neurons organized in an input layer, one or more hidden layers and an output layer as shown in Figure 8. However, since aging data are inherently time-dependent and thus sequential, Recurrent Neural Network (RNN) architectures are more suitable to handle aging data and therefore more frequently employed (Figure 8). These networks feature iterative connections within neurons, enabling them to retain contextual information throughout a sequence. To address the vanishing gradient problem, which hinders long-term sequence learning, more sophisticated cell types have been developed. Long Short-Term Memory (LSTM) cells were introduced in 1997 [114], while reference [115] proposed a lighter variant called Gate Recurrent Unit (GRU) in 2014. Both LSTM [13,116,117,118,119,120,121,122,123,124,125,126,127,128,129,130,131,132,133] and GRU [119,134,135,136,137] cells are now quite systematically used in recurrent architectures for prognosis modeling. Bidirectional GRU (biGRU) [106,138] and Bidirectional LSTM (biLSTM) [101,139,140] networks are also applied. The latter models process sequences in both directions, capturing past and future context, unlike standard RNNs which only move forward. Moreover, GRU and LSTM architectures remain flexible enough to be customized for specific needs. For instance reference [137] proposes a modified GRU model, the Gate-Aware GRU, to better capture abrupt variations in the on-state voltage of a power device, symptomatic of bond wire lift-off events. In this model, the difference between consecutive sequence elements is used as a gate adjustment factor, thus improving sensitivity to abrupt temporal changes. With the introduction in 2017 of the Attention Principle [141], a new evolution of recurrent models emerged. Attention-based structures [142,143,144,145,146,147,148] integrate mechanisms that dynamically assign weights to elements within a sequence, allowing the model to capture complex degradation patterns. However, this is achieved at the cost of increased architectural complexity and computational demand. In parallel, Convolutional Neural Networks (CNN) are also widely used [118,119,121,147,149]. Their structure, shown in Figure 8, relies on convolutional filters to extract deeper representations from raw sequential data. CNNs are often used as the input layer of another neural network structure to enhance the quality of the temporal feature extractions. Reference [150] proposed to use reinforcement learning concepts through a Deep Deterministic Policy Gradient (DDPG). Reinforcement learning is a machine learning approach where an agent learns to make decisions by interacting with an environment and receiving feedback in the form of rewards or penalties. The presented Deep Architectures offer high performance for complex datasets though they demand significant computational resources. Several studies also explore Extreme Learning Machines (ELM) [142,151], which are single-hidden-layer FNN trained using the approach introduced in [152]. In ELMs, hidden layer weights are randomly initialized, and output weights are computed analytically. These architectures are lightweight and offer rapid training but tend to be less accurate on complex or noisy datasets.

Among non-Bayesian parametric methods, regression algorithms [53,124,153] and concepts borrowed from time series analysis, such as Grey models [154,155] and ARIMA/SARIMA [156,157] can be found. Regression methods involve algorithms that estimate the optimal parameters of a degradation model using statistical principles or optimization techniques. The drawback of these methods is their sensitivity to noise. Grey models are mathematical frameworks designed to model and predict system behavior under conditions of limited or uncertain information. They estimate the future evolution of a variable from the cumulative sum of known observations $x=\{x_{1},\;x_{1}+x_{2},\ldots ,\;x_{1}+\ldots +x_{n}\}$ and a simplified state space model $\frac{dx}{dt}=f\left(x\right)$ representing the underlying system dynamics. They are particularly effective when data are limited in size but may exhibit reduced performance when facing complex or highly nonlinear degradation behaviors. The ARIMA (AutoRegressive Integrated Moving Average) model is used to model non-stationary time series. It relies on differencing the series to render it stationary, then models future values as linear combinations of past values (autoregression) and past errors (moving average). The SARIMA (Seasonal ARIMA) model extends this concept by incorporating a seasonal component.

Among parametric methods derived from Bayesian theory, Maximum Likelihood Estimation (MLE) [100,158,159] and Kalman Filters (KF) based algorithms [100,133,138,160,161,162] can be found. MLE is a statistical principle that estimates the parameters of a stochastic model by maximizing the likelihood function, thereby identifying the values that make the observed data most probable. Like regression methods, the drawback of MLE is its sensitivity to noise. Filtering methods address the noise sensitivity issues. The KF is a recursive estimation algorithm for linear dynamic systems affected by Gaussian noise. It works in two steps: predicting the next state and updating it using new data to minimize error. The Extended Kalman Filter (EKF) in [162] adapts this concept to nonlinear systems through local linearization. These filters are efficient and computationally fast, provided that the process and measurement noises are Gaussian. However, in practice, the accurate estimation of the model and the measurement noise remains challenging and critically affects the performance of the filter. The Particle Filter (PF) [101,154,161,162,163,164,165,166,167] is a semi-parametric extension of the Kalman filter applicable to non-linear and non-Gaussian systems. System’s states are represented as weighted random samples called particles, each corresponding to a possible hypothesis of the system’s state. These particles are iteratively propagated and resampled based on their consistency with observations. Although highly general, PFs are computationally demanding, with performance depending on both the number of particles and the resampling strategy. Among semi-parametric methods, the Gaussian Process Regressor (GPR) [104,150,168,169,170] can also be found. The GPR is a probabilistic regression technique that models observed points as noisy realization of an underlaying function drawn from a Gaussian process. This Gaussian process is defined by a mean and a covariance (kernel) function. The choice of kernel encodes assumptions about the smoothness, periodicity, or other structural properties of the target function, enabling the model to capture complex, non-linear dependencies. The optimization of hyperparameters for this method can be tedious.

Other algorithms traditionally used for prognosis exist. However, they are not included in Figure 7, as they cannot, or only rarely, be used independently to construct a prognosis tool. They are thus generally employed as complements to the algorithms previously discussed. For instance, correlation analysis methods [13,109,110,111,112,118,145], information fusion [109,111,112,118] or noise reduction methods can be mentioned. Considering information fusion, the Principal Component Analysis (PCA) [109,111,112,118] algorithm frequently appears in the literature. PCA is a statistical method for dimensionality reduction that converts the variables of a dataset into a new set of uncorrelated variables, thereby capturing the largest portion of the variance, and thus the information contained in the initial dataset. In the context of noise reduction, the predominant approaches consist of moving-average methods, implemented with different degrees of complexity and refinement [109,116,120,132,167], ranging from simple moving averages to polynomial smoothing methods. Reference [144] uses a noise reduction method based on wavelets. Adaptive signal decomposition methods are also encountered, such as Empirical Mode Decomposition (EMD) [123,126], Variational Mode Decomposition (VMD) [101,127,139,143], Trend Residual Decomposition (TRD) [137] or Complete Ensemble Empirical Mode Decomposition with Adaptive Noise (CEEMDAN) [136,146]. Temporal alignment concepts can also be mentioned [136], such as Dynamic Time Warping (DTW), which measures the similarity between two sequences by locally stretching or compressing time to measure the cumulative distance. This alignment minimizes the cumulative distance between corresponding points of the sequences.

#### 3.2.3. Implementation

**Figure 9:**
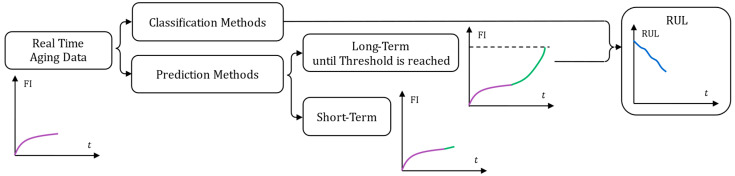
Methodological classification of Data-Based prognosis algorithms identified in the literature (FI: failure indicator).

In terms of implementation, the authors propose the methodological classification illustrated in Figure 9 for Data-Based prognosis methods reported in the literature.

Two main categories can be distinguished: classification methods and prediction methods. Classification methods aim to directly estimate the RUL of a component from measurements of degradation indicators. Prediction methods aim to forecast the future evolution of degradation indicators or advanced representations such as the Health Index. Prediction methods can be divided into two types: those that forecast short-term changes in a degradation indicator over a fixed time horizon, and those that model its progression until a failure threshold is reached, from which RUL is estimated. Although both approaches fall under the general framework of prognosis, the type of information they provide directly influences the predictive maintenance strategy that can be implemented. Methods that provide an explicit RUL estimate, either through classification or until-failure prediction, enable a global assessment of the component’s health status, thus supporting long-term maintenance planning. Conversely, short-term prediction methods require maintenance to be organized over a fixed time horizon, aligning more closely with condition-based maintenance rather than predictive maintenance. These methods are typically developed using accelerated aging datasets, particularly for classification-based approaches. Prediction methods, especially short-term ones, can alternatively be trained solely using historical degradation data collected directly from the monitored component. This enables the prognosis tool to capture the specific degradation behavior of the component under observation, thereby reducing potential biases associated with training datasets generated under different aging conditions.

Classification methods primarily rely on alignment techniques or machine learning algorithms such as SVM, Decision Trees, Random Forests, or neural networks, whereas prediction methods may employ any of the algorithms presented in Figure 7. Moreover, regardless of the chosen approach, the implementation of a prognosis tool often involves combining multiple algorithms, independently from the classification proposed in Figure 7, with the aim of leveraging the complementary strengths of each algorithm.

**Figure 10:**
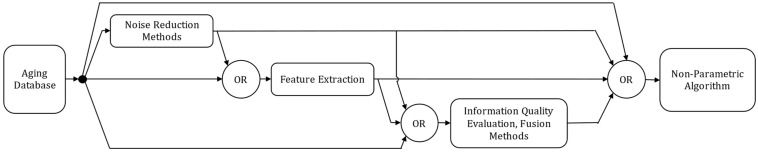
Tentative schematic overview of the implementation of a non-parametric method.

Regarding non-parametric methods, whether for prediction or classification, the general implementation framework is illustrated in Figure 10, based on trends identified in the literature.

Most studies focus on neural network-based approaches, where the main effort is devoted to data preprocessing. Given the available data, the aim is to extract as much information as possible that accurately characterizes the health state of the component under scrutiny and its evolution over time. The literature reports mostly single-indicator approaches, though multi-indicator structures could be used [104,106,118,131,138,145]. Multi-indicator methods enhance the amount of information provided to the algorithm, generally leading to improved inference performance. However, it requires a more sophisticated measurement set up for the device monitoring which can be challenging at an industrial scale. Not to mention the intrusiveness of the measurement scheme, which could affect the intrinsic reliability of the device. The selected measurement(s) can directly be considered as learning feature(s). However, three recurring challenges are typically reported: noise measurement, the amount of available information and the quality or redundancy of this information. The latter issues can limit the algorithm learning efficiency and generalization capabilities. To address noise issues, researchers frequently apply smoothing techniques such as moving average filters [109,116,120,132,167], adaptive signal decomposition methods [123,126,127,136,137,139,143,146] or wavelet noise reduction [144] to extract the underlying degradation trends. To enrich the information content, several authors propose computing new features derived from the original measurements. Many works extract temporal statistical indicators such as kurtosis or variance [106,111,112,118,145,168], others derive features from frequency-domain representations [117,135], while some authors directly use information obtained from adaptive decomposition as input features [126,137,143]. Finally, to mitigate redundancy and improve information relevance, several studies employ correlation analysis or entropy-based feature selection to retain only the most significant features [13,109,110,112,118,145,168], while others implement fusion techniques such as PCA [109,111,112,118]. These sequential features are then segmented using a sliding-window approach to construct the training dataset.

Non-parametric prediction methods can be implemented in two modes: single-step or multi-step ahead [171], operating either in open-loop or closed-loop configuration [135]. Given a sequence of past data up to time t, single-step and multi-step ahead prediction modes, respectively, generate estimates at horizons t+1 and t+n (with n>1). In the open-loop configuration, predictions rely solely on measured data, thus limiting the forecast horizon to short-term predictions. In the closed-loop configuration, the predicted data are iteratively fed back into the model input, enabling longer-term forecasts up to a defined degradation threshold. However, this approach inherently leads to error accumulation as iterations progress. The choice of prediction mode represents a key aspect of the prognosis algorithm design. Nevertheless, many studies lack clarity regarding the prediction configuration they employ, thus making it difficult to classify the prognosis method between short-term and until-threshold prediction. Neural network-based prediction methods also require large amounts of training data. Consequently, when models are built exclusively on historical degradation data of the monitored component, their predictive accuracy tends to be reliable only during the mid to late stages of the component’s life. This limitation can hinder the effective planning and organization of maintenance activities.

**Figure 11:**
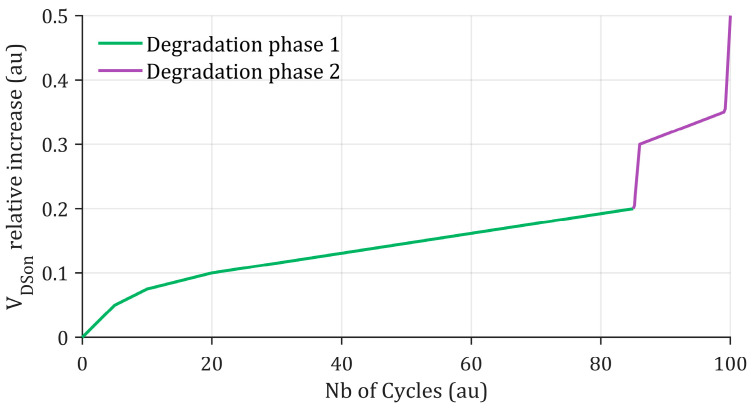
Adaptation of the degradation model and/or algorithm across different degradation phases.

Regarding parametric methods, including PFs, the main challenge lies in the selection of an appropriate degradation model. Given the highly nonlinear variations which may typically be observed in degradation indicators, such as the on-state voltage, several studies propose segmenting the prediction domain of their prognosis tools [100,154,155,163]. In these approaches, the degradation model and/or the underlying algorithm are adapted for each phase of the degradation process, as illustrated in Figure 11.

For instance, ref. [155] proposes the use of variable-step GM depending on the degradation stage. Similarly, ref. [100] employs a Wiener process in which the degradation model evolves according to the aging progression. Another study [154] suggests modifying both the algorithm and the degradation model, modeling the early degradation phase with a Verhulst GM and the later phase with a PF based on a double-Gaussian degradation model. Ref. [153] proposes an empirical degradation model that is iteratively refitted via regression each time a bond wire lift off is detected. These variable-dynamic architectures aim to adapt to abrupt variations that may occur in degradation indicators, thereby improving estimation accuracy. However, they generally fail to predict such abrupt changes over long time horizons, restricting their applicability most of the time to short-term prognosis. Regarding GPR, ref. [170] proposes an Optimal Scale GPR approach. The main idea is to dynamically adjust the correlation interval length to help the model better capture abrupt variations in the degradation indicators.

Some studies aim to integrate Bayesian principles into non-Bayesian methods, primarily to enable uncertainty quantification in predictions or classifications. Several papers introduce the concept of Mixture Density Neural Networks (MDNN) [13,128]. Instead of training a network to predict a single target value, an MDNN is trained to predict the parameters of a probability density function whose likelihood is maximized at the target value. Within this framework, the MDNN constitutes the output layer of the prognostic neural network, enabling the assessment of a confidence level for each prediction. In [137], the authors propose to train a dedicated neural network for uncertainty estimation, based on statistical considerations rather than probabilistic modeling. The authors in [145] use Monte Carlo drop out to evaluate the uncertainty of neural network prediction. The authors in [103,167] propose to combine the output of their non Bayesian model with a PF and thus model the uncertainty of their model’s prediction through the PF particles.

Other works explore more advanced algorithmic combinations [101,136]. For instance, reference [136] combines alignment-based methods with recurrent neural networks and stochastic processes, leveraging the strengths of each approach to enhance prognosis robustness and reliability.

#### 3.2.4. Advanced Optimization Concepts

Other studies have focused on advanced optimization strategies designed to improve both the performance and robustness of prognostic algorithms. Many authors have explored the implementation of metaheuristic optimization algorithms inspired by natural phenomena. Regarding hyperparameter optimization, ref. [105] employs a Crested Porcupine Optimizer to tune the hyperparameters of an SVM, while [108] uses an improved Dung Beetle Algorithm to optimize those of an FNN. Similarly, ref. [139] applies a Pelican Optimization Algorithm to optimize the hyperparameters of an RNN, and refs. [132,151] utilize a Whale Optimization Algorithm to, respectively, improve the learning of an ELM and tune an RNN. Ref. [170] implements an Ant Lion Optimizer to dynamically adjust the correlation interval length in an Optimal Scale GPR model. For PF optimization, ref. [164] applies Particle Swarm Optimization, while refs. [161,165] adopt genetic algorithms to improve the resampling process of PF particles.

Other optimization ideas could be found. In [135], a Scheduled Teaching and Professor Forcing training strategy is implemented to improve the closed-loop prediction accuracy of RNNs. However, enhancing the generalization capabilities of models has remained one of the most significant research directions in recent years. These optimization approaches are primarily applied within the field of neural network-based prognosis, encompassing studies focused on dataset augmentation [13] and Transfer Learning techniques [136,145,149,172].

Ref. [13] enhances the accelerated aging dataset through a synthetic data generation approach known as the Conditional Tabular Generative Adversarial Network (CTGAN). CTGAN is an adversarial learning model designed to generate realistic synthetic tabular data. It consists of two main components: a generator, which produces synthetic samples based on the statistical distribution of the training dataset, and a discriminator, which attempts to distinguish real data from synthetic one. During training, these two modules are optimized in a competitive process until the generator can produce synthetic data that the discriminator can no longer differentiate from real samples. CTGAN introduces mathematical mechanisms to ensure the global statistical consistency of the generated dataset, thereby preserving the structural and statistical properties of the original data distribution.

Transfer Learning methods, also known as Domain Adaptation, are optimization techniques designed to improve the performance of a neural network trained on a source domain when applied to a target domain. In this context, the source domain consists of an accelerated aging dataset, while the target domain contains actual aging data of the component under prognosis evaluation. By definition, the source domain provides a large, well-labeled dataset, whereas the target domain offers a significantly smaller one. Reference [172] proposes a Weakly Supervised Adversarial Training (WSAT) method, based on Weakly Supervised Domain Adaptation (WSDA). The objective of WSDA is to align the data distributions of the source and target domains, allowing the model to effectively leverage knowledge from the source domain to improve performance on the target domain. In WSAT, this domain alignment is achieved through adversarial training. Other works [136,145] employ Transfer Learning methods based on network fine-tuning, a concept popularized by large language models. The approach consists of initially training a network on a generic, large-scale source dataset, and then refining its training to adapt to the specific requirements of the target domain. For instance, ref. [136] trains a neural network on a dataset of various aging indicator curves. The network is then fine-tuned using real aging data from the target component, selecting source curves that are closely similar to the behavior of the measured target component.

### 3.3. Hybrid Methods

#### 3.3.1. General Considerations

Hybrid methods combine concepts derived from both Physics-Based and Data-Based approaches, with the objective of leveraging the advantages of each. Data-Based methods, through their algorithmic frameworks, can compensate for the incomplete physical modeling of Physics-Based approaches and thus strengthen their predictive capabilities. Conversely, incorporating physical principles into a Data-Based method can guide the learning process of the algorithm, reducing the amount of training data required and improving the interpretability of the resulting model.

The degree of hybridization in a prognosis method can vary significantly, ranging from a simple inclusion of physics-informed features within a neural network learning process to more sophisticated frameworks that deeply integrate both Data-Based and Physics-Based concepts. Accordingly, the authors propose to distinguish between light hybridization and strong hybridization. The following section presents the latest advances and trends reported in the literature.

#### 3.3.2. Light Hybridization

Light hybridization prognosis methods introduce physical principles into Data-Based approaches or, conversely, employ Data-Based algorithms to support Physics-Based methods, without establishing a deeply integrated connection between the two.

For Data-Based methods enhanced by physical concepts, one notable example is the Physics-Informed Neural Network (PINN). PINNs are neural networks whose learning process and/or architecture are constrained by physical laws. The loss function of such networks can therefore be expressed by Equation (7):

(7) $$Loss(y_{true},\;y_{pred})\;=\;f_{error}(y_{true},\;y_{pred})\;+\;f_{physical}(y_{pred})$$

This loss function evaluates the error between the true target value and its prediction, while penalizing predictions that violate established physical principles. The implementation of this penalization can vary in complexity. In the context of light hybridization, penalization typically relies on simple physical constraints. For instance, ref. [130] proposes a Data-Based classification method using a RNN. As detailed by Equation (1), the RUL is bounded between 0 and 1 and decreases over time. Therefore, ref. [130] introduces a loss function that penalizes RUL predictions falling outside this range or violating this monotonicity criteria, thereby guiding the network’s learning process. Those physical constraints are found in [140] too. Similarly, ref. [129] develops a method for predicting variations in on-state voltage, which are also assumed to be monotonic. In this case, the authors penalize predictions whose first and second derivatives are non-positive during the training phase to improve the physical consistency of the model outputs.

Regarding Physic-Based methods supported by algorithms from Data-Based concepts, one relevant example is the use of machine learning algorithms as surrogates of FEM simulations. As discussed in Section 3.1.2, the stress quantities used in physical lifetime model approaches are typically computed through FEM simulations based on the component’s operating conditions. However, such simulations require significant computational resources, time and detailed knowledge of the component architecture. To address this issue, authors [102,173] propose training a machine learning algorithm from FEM simulation results. The underlying idea is to leverage the generalization capabilities and computational efficiency during inference of machine learning algorithms to avoid time consuming FEM simulations at inference.

#### 3.3.3. Strong Hybridization

Prognosis methods based on strong hybridization generally involve a sophisticated integration of Physics-Based and Data-Based concepts. As a result, it is more difficult to identify common methodological trends, since each study typically introduces a novel contribution.

For example, ref. [122] proposes a classification method that accounts for the influence of the component’s ambient temperature on the acceleration of its degradation. Their approach includes an estimation of the component’s junction temperature. By using the measured ambient temperature, the authors compute the degradation acceleration factor according to the Arrhenius law, which is then incorporated as an input feature into a neural network. Reference [158] proposes an until-threshold prediction method based on a stochastic model whose parameters are estimated via maximum likelihood, with the degradation model derived from Paris’ law describing crack propagation. Reference [173] introduces a complex until-threshold prediction framework. A GPR is used to evaluate the crack length of the chip-bond wire interface from the on-state voltage. An SVR, trained based on FEM simulation results, evaluates physical quantities like average contact stress of the crack length and usage conditions. Then, a Markov chain is implemented to evaluate the crack length evolution from the latter physical quantities and thus infer the variation in the on-state voltage. The process is iteratively repeated until the predicted on-state voltage reaches a threshold.

Finally, regarding PINNs, the physical penalization term in Equation (7) can be extended beyond simple boundary or monotonicity constraints [148,149]. For instance, ref. [149] proposes to compare the network’s predictions with those obtained from a Physic-Based model and to penalize prediction deviations.

## 4. Discussion

The previous section provides an overview of the various methods and trends reported in the literature concerning the prognosis of power semiconductor devices for health state and RUL prediction, while highlighting common advantages and limitations associated with these approaches. The present section aims to take a step further by offering a critical perspective on methodological concepts and implementation feasibility aspects that are often underemphasized in existing studies.

Regarding Physics-Based approaches, the main challenges lie in accurately estimating stress profiles and understanding how the sequencing of stress cycles influences device lifetime. Estimating stress profiles, whether arising from direct or indirect physical constraints, is a complex task and strongly depends on the device’s wear-out state. Therefore, the reliable measurement or reconstruction of these stresses over the entire device lifetime is still an active area of research. For instance, this difficulty is illustrated by the extensive body of literature devoted to estimating the junction temperature of active power components through direct or indirect measurement methods [174,175,176,177] as well as by more recent studies that investigate the drift of such indirect measurement methods induced by device aging [178,179,180,181]. In addition, lifetime models often rely on simplifying assumptions that significantly limit the robustness of PoF-based predictions, particularly with respect to the impact of stress cycles sequencing to the device lifetime. For instance, the authors in [182,183] analyze the influence of temperature cycling sequence order on component lifetime and report results that contradict the assumptions underlying Miner’s rule, which is based on linear damage accumulation. As a consequence, most Physics-Based approaches exhibit a limited range of applicability, constrained by the validity of their underlying modeling assumptions.

Data-Based methods face their own challenges too. Regarding prediction methods, their performance and applicability are strongly conditioned by the behavior of the degradation indicators they aim to forecast. Indeed, predicting a smooth and monotonic degradation trend, as illustrated in Figure 5—Shape B, is considerably less challenging than forecasting abrupt or irregular variations, as shown in Figure 5—Shape A. Furthermore, a growing number of recent studies employ increasingly sophisticated neural network architectures, justifying their superiority over simpler models by their ability to capture high-frequency variations in degradation indicators. However, the information most relevant to assess a component’s health state resides primarily in its long-term degradation trend rather than in high-frequency fluctuations, which raises questions about the necessity and practical value of such complex architectures. A major limitation of Data-Based methods concerns their ability to perform out-of-sample predictions. Most studies validate the performance of their prognosis tools using components that have undergone the same degradation process as the ones represented in the training data, what does not demonstrate the method’s ability to generalize to complex real-world operating conditions. Those validations only act as proof of concept: if the algorithm is trained using devices subjected to real-world aging conditions, it should indeed perform effectively on other devices exposed to the same stresses. Nevertheless, as discussed in Section 2.1, the expected lifetime of most active power components, such as SiC MOSFETs, extends over several decades. Considering this timescale, together with the continuous technological advancements in SiC MOSFETs, developing prognosis tools based on real aging data is generally impractical. Therefore, greater attention should be given to studies such as [13,119,173] which attempt to evaluate the performance of their prognosis model to extrapolated aging conditions. In addition, the practical measurability of degradation indicators, as discussed in [163], remains insufficiently addressed in many studies. Although numerous indicators exhibit strong sensitivity or favorable linearity with respect to underlying degradation mechanisms, their accurate online measurement in industrial environments remains highly challenging.

Beyond methodological considerations, implementation aspects are also often overlooked. While many industrial systems rely on centralized Supervisory Control And Data Acquisition (SCADA) architectures, a wide range of applications require embedded, real-time prognosis capabilities. This requirement raises important concerns regarding computational complexity, resource constraints, and the practical feasibility of deploying advanced prognosis algorithms. Finally, a critical gap persists across nearly all prognosis approaches: the almost systematic absence of uncertainty quantification. As highlighted in [184], RUL estimation is inherently affected by multiple sources of uncertainty, and explicit confidence bounds are essential for enabling reliable RUL predictions that can effectively support predictive maintenance decision-making. Although RUL prediction exhibits application-dependent characteristics and constraints, uncertainty estimation constitutes a general challenge inherent to all prognosis methods regardless of the specific application domain. While several elements related to uncertainty quantification have been discussed throughout this paper, this topic is considerably broader and is addressed in greater depth in dedicated studies such as [184].

In light of the aforementioned limitations and based on the body of reviewed literature, current RUL prognosis methods for SiC MOSFETs appear to still lack the level of robustness required to safely support their deployment within industrial predictive maintenance strategies.

## 5. Conclusions

This article presents a comprehensive literature review on prognosis methods for SiC MOSFETs, highlighting methodological trends and current limitations. The prerequisites for designing an effective prognosis tool are discussed in detail. The assumptions underlying accelerated aging experiments for SiC MOSFETs can significantly constrain the applicability of the developed prognosis methods. Research articles published up to 2025 proposing prognosis techniques for power semiconductor devices were identified through OpenAlex and manually filtered according to the scope of this study. The selected approaches were classified into three main categories: Physics-Based, Data-Based, and Hybrid methods. Physics-Based methods primarily rely on PoF approaches. The extraction of stress profiles and the understanding of the impact of stress sequencing on component aging remain the major limitations to the robustness of Physics-Based prognosis tools. Data-Based methods represent most of the reviewed works, with a significant proportion relying on neural networks. From a methodological framework, these approaches can be classified into two categories: classification methods and short-term or until-threshold prediction methods. The performance of such methods is highly dependent on the evolution patterns of degradation indicators, while validation through out-of-sample testing remains unfortunately uncommon. Hybrid methods have recently gained increasing attention in literature. Depending on the degree of integration, they aim to mitigate the shortcomings of data-based methods by incorporating physics-based principles, and conversely. Overall, too few studies address the evaluation of uncertainty associated with prognosis predictions, which is essential for the reliable implementation of predictive maintenance strategies. Despite significant methodological and algorithmic progress, ensuring the reliability of prognosis tools for SiC MOSFETs remains an open research challenge so far.

## Figures and Tables

**Table 1:** Summary of the main SiC MOSFET failure mechanisms, acceleration factors, and aging indicators.

Failure Location	Cause	Acceleration Factors	Failure Indicators
Gate Oxide	Carrier tunneling	High Electric Field, High Temperature	$V_{th}$: Threshold voltage
			$I_{gss}$: Gate leakage current
			$R_{on}$$,\;V_{on}$: on state resistance, voltage
			$V_{gp}$: Plateau Miller voltage
			$T_{on}$$,\;T_{off}$: turn on, off duration
Body Diode	Energy released by the carrier recombination at PN junction	Forward current	$V_{f}$: forward body diode voltage
			$I_{dss}$: Drain leakage current
Bond Wires	Thermomechanical stress	Fast temperature cycling	$R_{on}$$,\;V_{on}$: on state resistance, voltage
			$V_{f}$: forward body diode voltage
Solder Layers		Slow temperature cycling	$R_{th}$: thermal resistance junction to baseplate

**Table 2:** Summary of traditional lifetime model used in literature.

Model Name	Model Expression	Parameter	Variable
Coffin–Manson [64,73]	$N_{f}=a_{1}\Delta T_{j}^{a_{2}}$	$a_{i}$ (i = 1,2)	$\mathrm{Junction}\;\mathrm{temperature}\;\mathrm{swing}\;(\Delta T_{j})$
Coffin–Manson-Arrhenius (LESIT) [62,67,76]	$N_{f}=a_{1}\Delta T_{j}^{a_{2}}\exp\left(\frac{E_{a}}{kT_{m}}\right)$	$a_{i}$ ($i\;=\;1,2),\;E_{a}$ activation energy, k Boltzmann constant	$\Delta T_{j}$, $\mathrm{mean}\;\mathrm{junction}\;\mathrm{temperature}\;(T_{m})$
Norris–Landzberg [74]	$N_{f}=a_{1}\Delta T_{j}^{a_{2}}f^{a_{3}}\exp\left(\frac{E_{a}}{kT_{m}}\right)$	$a_{i}$ $(i\;=\;1\ldots 3),\;E_{a}$, k	$\Delta T_{j}$, $T_{m}$, frequency (f)
Bayerer (CIPS2008) [58,61,65,75]	$N_{f}=a_{1}\Delta T_{j}^{a_{2}}t_{on}^{a_{3}}I_{B}^{a_{4}}V_{c}^{a_{5}}D^{a_{6}}\exp\left(\frac{a_{7}}{T_{j_{min}}+273}\right)$	$a_{i}$ (i = 1…7)	$\Delta T_{j}$, $\mathrm{heating}\;\mathrm{time}\;(t_{on}),\;\mathrm{current}\;\mathrm{per}\;\mathrm{bond}\;\mathrm{foot}\;(I_{B}),\;\mathrm{voltage}\;\mathrm{class}\;(V_{C}),\;\mathrm{bond}\;\mathrm{wire}\;\mathrm{diameter}\;(D),\;\mathrm{minimum}\;\mathrm{junction}\;\mathrm{temperature}\;(T_{j_{min}}),$

## Data Availability

No new data were created or analyzed in this study.

## Abbreviations

The following abbreviations are used in this manuscript:

CNN	Convolutional Neural Network
CTE	Coefficient of Thermal Expansion
CTGAN	Conditional Tabular Generative Adversarial Network
DBC	Direct Bounded Copper
EKF	Extended Kalman Filter
ELM	Extreme Learning Machine
FEM	Finite Element Modeling
FNN	Feedforward Neural Network
GM	Grey Model
GPR	Gaussian Process Regressor
GRU	Gate Recurrent Unit
IGBT	Insulated Gate Bipolar Transistor
KF	Kalman Filter
LSTM	Long Short-Term Memory
MDNN	Mixture Density Neural Network
MLE	Maximum Likelihood Estimation
MOSFET	Metal Oxide Semiconductor Field Effect Transistor
PCA	Principal Component Analysis
PCoE	Prognostics Center of Excellence
PF	Particle Filter
PINN	Physics-Informed Neural Network
PoF	Physics of Failure
RNN	Recurrent Neural Network
RUL	Remaining Useful Life
SCADA	Supervision Control And Data Acquisition
SOA	Safe Operating Area
SVM	Support Vector Machine
WBG	Wide Band Gap
WSAT	Weakly Supervised Adversarial Training
WSDA	Weakly Supervised Domain Adaptation
